# Microparticles Released by Dengue Virus-Infected Monocytes Mediate Endothelial Activation and Vasculopathy

**DOI:** 10.3390/ijms27125367

**Published:** 2026-06-14

**Authors:** Janet García-Pillado, Pedro Pablo Martínez-Rojas, Elizabeth Quiroz-Garcia, Carlos Cabello-Gutiérrez, Marcela Lizano, Luis Padilla-Noriega, Lourdes Teresa Agredano-Moreno, Luis Felipe Jiménez-García, Blanca H. Ruiz-Ordaz

**Affiliations:** 1 Departamento de Biología Molecular y Biotecnología, Instituto de Investigaciones Biomédicas, Universidad Nacional Autónoma de México, Ciudad de México 04510, Mexico; janetgarcia@ciencias.unam.mx (J.G.-P.); pedropablo.martinezrojas@gmail.com (P.P.M.-R.); geliqg@gmail.com (E.Q.-G.); 2 Departamento de Investigación en Virología y Micología, Instituto Nacional de Enfermedades Respiratorias “Ismael Cosío Villegas”, Ciudad de México 14080, Mexico; carloscginer@gmail.com; 3 Departamento de Medicina Genómica y Toxicología Ambiental, Instituto de Investigaciones Biomédicas, Universidad Nacional Autónoma de México, Ciudad de México 04510, Mexico; lizano@unam.mx; 4 Departamento de Microbiología y Parasitología, Facultad de Medicina, Universidad Nacional Autónoma de México, Ciudad de México 04510, Mexico; lpadilla@unam.mx; 5 Departamento de Biología Celular, Facultad de Ciencias, Universidad Nacional Autónoma de México, Ciudad de México 04510, Mexico; agredano-moreno@ciencias.unam.mx (L.T.A.-M.); luisfelipe_jimenez@ciencias.unam.mx (L.F.J.-G.)

**Keywords:** dengue, dengue virus, severe dengue, monocytes, endothelial vascular cells, extracellular vesicles, microparticles, microparticles mediating viral dissemination, procoagulant and proinflammatory phenotype, vasculopathy

## Abstract

Dengue is the most prevalent arthropod-borne viral disease, caused by infection with the dengue virus (DENV). Severe dengue is characterized by significant vasculopathy involving a proinflammatory and procoagulant state associated with increased vascular permeability. However, the host–virus interactions driving this process remain incompletely elucidated. Monocytes (Mø) are primary target cells during DENV infection and actively release extracellular vesicles, like microparticles (MPs), mediating intercellular communication, contributing to dengue pathogenesis. Here, we evaluated whether MPs released by DENV-infected monocytes represent a previously underappreciated mechanism contributing to dengue-associated vascular dysfunction. The vascular endothelium plays a determining role in the response to injury because it functions as a regulatory interface during hemostasis (coagulation–fibrinolysis–inflammation) and by preserving the endothelial barrier. We found that these vesicles transport viral proteins (E and NS1), exhibit a procoagulant profile that promotes thrombin generation, and enhance endothelial vascular cell (EVC) activation. DENV-infected THP-1 Mø MPs interaction induces a shift toward a procoagulant, proinflammatory, and proadherent phenotype, characterized by increased expression of PAR-1, TF, ICAM-1, and VCAM-1, reflecting the establishment of a sustained HMEC-1 EVC activation that compromises vascular barrier integrity. This leads to increased permeability, a hallmark of DENV-associated vasculopathy and a central event in the progression to severe dengue.

## 1. Introduction

Dengue, also referred to as dengue fever, is an acute febrile and systemic mosquito-borne viral disease caused by infection with any of the four antigenically distinct dengue virus (DENV) serotypes. DENV is a positive-sense, single-stranded RNA virus that belongs to the genus *Orthoflavivirus* within the *Flaviviridae* family [1,2]. According to the World Health Organization, approximately 50% of the global population is currently at risk of DENV infection, with an estimated 100–400 million infections occurring annually. Dengue is considered endemic in more than 130 countries worldwide [1].

Viral transmission occurs primarily through the bite of infected *Aedes* spp. mosquitoes, and infection can result in a broad spectrum of clinical manifestations, ranging from asymptomatic or mild illness to severe dengue (SD), previously known as dengue shock syndrome and dengue hemorrhagic fever. SD may involve plasma leakage, hemorrhage, organ impairment, and potentially fatal outcomes [3,4].

The pathogenesis of SD is characterized by high levels of viremia associated with inefficient viral clearance, together with marked activation of DENV target cells such as monocytes [5,6,7], macrophages, dendritic cells [8,9], and platelets [10,11], leading to increased production of proinflammatory cytokines. These events contribute to hemostatic disturbances, including thrombocytopenia, dysregulation of the coagulation–inflammation systems, and vascular damage [12]. Consequently, interactions between DENV-infected cells and vascular endothelial cells disrupt the endothelial basal state—characterized by anticoagulant and anti-inflammatory functions and preserved barrier integrity—promoting a vasculopathy defined by a procoagulant and proinflammatory phenotype with increased vascular permeability and plasma extravasation [13].

Monocytes (Mø) function as key regulators of the host response by contributing to viremia control while simultaneously acting as central drivers of immunopathology. Beyond their antiviral role, activated and DENV-infected monocytes can amplify inflammatory signaling, facilitate antibody-dependent enhancement of infection, and promote endothelial dysfunction, processes described in SD [5,6]. Accordingly, monocytes respond to DENV infection by becoming activated and undergoing differentiation [7,14,15], leading to the expression of multiple bioactive products, including inflammatory mediators [16], coagulation-associated proteins such as Tissue Factor (CD142) [17,18], and the release of extracellular vesicles (EVs) [19].

Extracellular vesicles are cell-derived, lipid bilayer-enclosed particles released into the extracellular milieu that lack autonomous replicative capacity [20]. Functionally, EVs are increasingly recognized as active mediators of intercellular communication, capable of transferring bioactive cargo that modulates inflammatory, immune, and vascular responses, thereby contributing to both homeostatic regulation and disease pathogenesis [21]. Based on their biogenesis, EVs are broadly classified into small EVs, commonly referred to as exosomes, which originate from the endosomal pathway, and large EVs, also termed ectosomes, microparticles, or microvesicles, which are generated by direct outward budding from the plasma membrane [22,23].

Microparticles (MPs) are increasingly recognized as key players in the pathogenesis of vascular diseases associated with chronic inflammation, endothelial damage, and thrombosis [24,25]. MPs are released by multiple cell types, with platelet-derived MPs representing the most abundant population in circulation and being primarily associated with procoagulant activity. During DENV infection, platelet activation drives the release of MPs that contribute to inflammation and endothelial dysfunction, both of which are tightly associated with disease severity [26]. Hottz et al. (2013) demonstrated that platelet-derived MPs, generated via mitochondrial oxidative signaling and inflammasome activation, increase endothelial permeability and correlate with plasma leakage and elevated hematocrit levels in severe dengue, supporting their value as biomarkers of disease progression [27]. Other reports have shown that MPs released from erythrocytes and platelets frequently expose phosphatidylserine and viral antigens: elevated erythrocyte MPs correlate with disease severity and complement activation, while reduced platelet MPs are associated with bleeding manifestations linked to thrombocytopenia [28,29].

However, the functional contribution of monocyte-derived MPs released during DENV infection to endothelial injury and barrier dysfunction remains poorly understood. Likewise, we previously reported that during in vitro Zika virus (ZIKV) infection, activated intermediate Mø release exosomes that carry viral components as part of their cargo. The naïve-cell–exosomes interaction promotes viral transmission, infection, and cell differentiation/activation. Hence, exosomes derived from ZIKV-infected Mø are an efficient alternative transmission route that may contribute to disease progression [30].

Based on the evidence supporting a role for MPs in dengue-associated vascular dysfunction, we hypothesized that MPs released by DENV-infected monocytes contribute to endothelial injury, barrier disruption, and the possible transfer of viral elements that promote viral dissemination. Therefore, the aim of this study was to investigate the contribution of monocyte-derived MPs to viral dissemination, endothelial activation, and vasculopathy associated with DENV infection. To address this, we employed in vitro models of monocyte infection and endothelial cell stimulation to evaluate the impact of monocyte-derived MPs on vascular damage and loss of barrier permeability associated with the pathogenesis of SD.

## 2. Results

### 2.1. DENV Infection Induces Procoagulant and Proinflammatory Phenotype in THP-1 Monocytes

**Figure 1:**
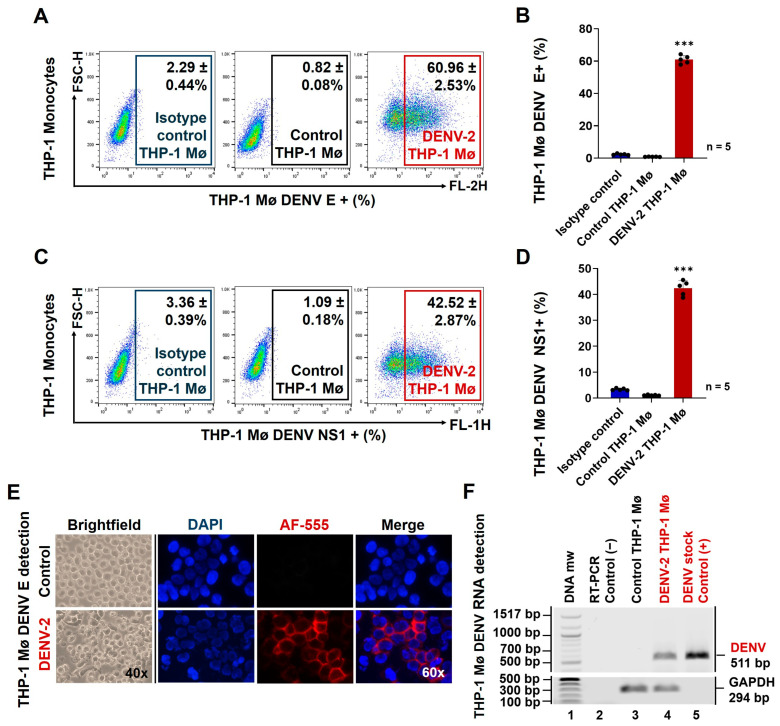
DENV-2 (MOI 1) infection in THP-1 Mø. (**A**) Detection of DENV E protein at 72 h p.i. (representative dot plots by FACS). (**B**) Percentages of E-positive THP-1 Mø. (**C**) Detection of DENV NS1 protein at 72 h p.i. (representative dot plots by FACS). (**D**) Percentages of NS1-positive THP-1 Mø. The percentages of THP-1 Mø E+ and NS1+ from DENV-2 THP-1 Mø were compared with Control THP-1 Mø values using an unpaired Student’s *t*-test. Statistical significance is denoted as *** when *p* < 0.0001. (**E**) Detection of the viral E protein (red) in DENV-infected THP-1 Mø by immunofluorescence microscopy (60×) at 72 h p.i. (**F**) Detection of DENV RNA by RT-PCR amplification in DENV-infected THP-1 Mø. Amplicons were visualized on 1.2% agarose gels stained with 2% ethidium bromide. Isotype control (blue), Control THP-1 Mø (black), and DENV-2 THP-1 Mø/DENV stock (red). n = 5 independent experiments.

First, we performed an in vitro infection of 1.0 × 10^6^ THP-1 monocytes (Mø) under previously established optimal conditions (MOI of 1, 72 h incubation at 37 °C, and 5% of CO_2_) and evaluated the presence of the viral envelope (E) and nonstructural 1 (NS1) proteins at the cell membrane surface. We found that 61% of DENV-exposed THP-1 Mø (DENV-2 THP-1 Mø) were positive for the viral E protein (*p* < 0.0001) (Figure 1A,B) and 43% were positive for the viral NS1 protein (*p* < 0.0001) (Figure 1C,D). These viral components were not detected in uninfected THP-1 cells (Control THP-1 Mø).

We confirmed the viral presence at 72 h p.i. through E protein immunostaining in DENV-infected THP-1 Mø by fluorescence microscopy (Figure 1E), and by viral RNA amplification (511 bp amplicon corresponding to the conserved C/prM genomic region) from infected THP-1 cell lysates (Figure 1F). A strong red signal associated with E protein expression in DENV-infected THP-1 cells was observed, which was absent in Control THP-1 Mø. In addition, the distinct DENV-specific amplicon band was detected in DENV-2 THP-1 Mø samples, but not in Control THP-1 Mø samples, indicating the presence of viral RNA within infected THP-1 monocytes. These results demonstrate that our experimental model supports productive DENV infection and viral replication in THP-1 monocytes, as evidenced by the detection of viral proteins and genomic RNA.

**Figure 2:**
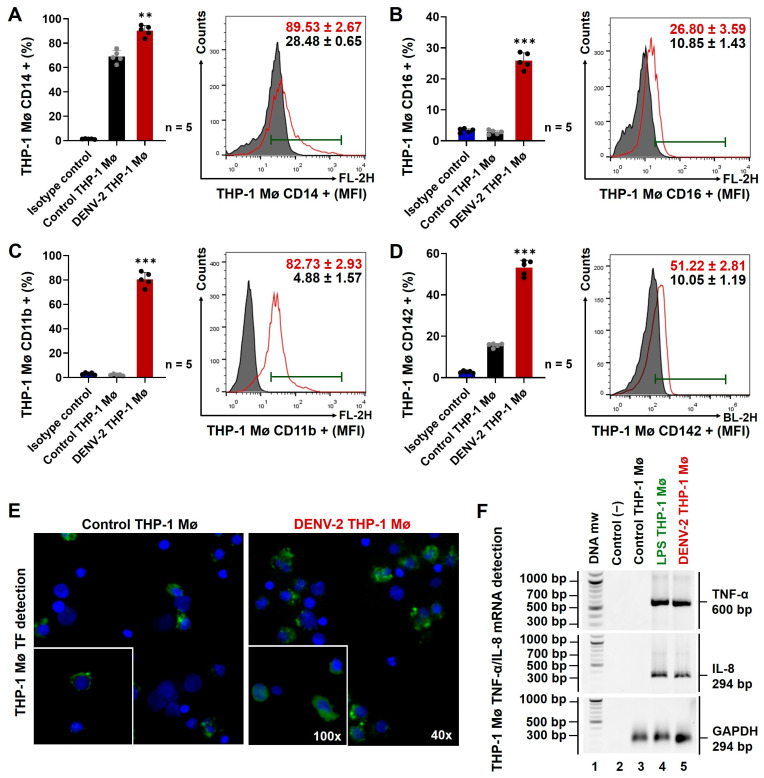
DENV infection promotes THP-1 monocyte differentiation and proinflammatory/procoagulant activation. (**A**) Percentage of CD14+ THP-1 Mø with a representative histogram showing mean fluorescence intensity (MFI) values obtained by FACS at 72 h p.i. (**B**) Percentage of CD16+ THP-1 Mø with a representative histogram showing MFI values obtained by FACS at 72 h p.i. (**C**) Percentage of CD11b+ THP-1 Mø with a representative histogram showing MFI values obtained by FACS at 72 h p.i. (**D**) Percentage of TF (CD142)+ THP-1 Mø with a representative histogram showing MFI values obtained by FACS at 72 h p.i. The percentages of CD14+, CD16+, CD11b+, and TF+ from DENV-2 THP-1 Mø were compared with Control THP-1 Mø using an unpaired Student’s *t*-test. Statistical significance is denoted as ** when *p* < 0.001 and *** when *p* < 0.0001. (**E**) Detection of TF (green) in THP-1 monocytes by immunofluorescence microscopy (40× and 100×) at 72 h p.i. (**F**) Detection of proinflammatory cytokine mRNA (TNF-α and IL-8) by RT–PCR amplification in THP-1 Mø. Amplicons were visualized on 1.2% agarose gels stained with 2% ethidium bromide. Isotype control (blue), Control THP-1 Mø (black), Lipopolysaccharide (LPS)-stimulated THP-1 Mø (dark green), and DENV-2 THP-1 Mø (red). n = 5 independent experiments.

Monocytes are recognized as primary target cells for infection by different orthoflaviviruses and exhibit different levels of activation and differentiation [5,6,7]. Here, we evaluated the levels of CD14 (lipopolysaccharide [LPS] receptor), CD16 (Fc gamma receptor III), CD11b (integrin alpha M), and CD142 (Tissue Factor [TF] or coagulation factor III), as well as the mRNA expression of proinflammatory cytokine (TNF-α and IL-8), to assess the potential proinflammatory response at the transcriptional level and procoagulant response (Figure 2).

Monocytes are classified into distinct populations based on CD14 and CD16 levels, namely classical, intermediate, and non-classical. Shifts in the distribution of these subsets reflect the establishment of an inflammatory state [6,7]. We evaluated CD14 and CD16 expression in response to DENV infection and found that, in uninfected THP-1 cells (Control THP-1 Mø), 69% (MFI = 28) and 3% (MFI = 11) of cells were CD14+ and CD16+ (Figure 2A,B), respectively, corresponding with the classical subset. In contrast, in DENV-infected THP-1 Mø (DENV-2 THP-1 Mø), 91% (MFI = 90) of cells were CD14+ and 26% (MFI = 27) were CD16+, indicating a shift toward the intermediate subset. The frequency of CD14+ and CD16+ monocytes increased by 1.3- (*p* < 0.01) and 9.3-fold (*p* < 0.0001), respectively, in DENV-2 THP-1 Mø compared with Control THP-1 Mø, suggesting that DENV infection promotes differentiation toward an intermediate, proinflammatory phenotype.

We also evaluated CD11b levels to further characterize THP-1 monocyte activation (Figure 2C) and found that 81% (MFI = 83) of DENV-2 THP-1 Mø were CD11b+ (*p* < 0.0001), whereas Control THP-1 Mø showed 2% (MFI = 5) of positivity, indicating that DENV infection enhances THP-1 monocyte activation. Elevated CD11b is associated with increased monocyte adhesion capacity, facilitating interaction with the endothelial vascular cells [31].

Given the enhanced activation and adhesion profile of DENV-infected THP-1 Mø, particularly the expansion of activated intermediate monocytes, we assessed whether this phenotype is associated with the upregulation of TF, a key initiator of the coagulation cascade. DENV infection increased TF levels in THP-1 monocytes by approximately 3-fold, from 16% (MFI = 10) in Control THP-1 Mø to 53% (MFI = 51) in DENV-2 THP-1 Mø (*p* < 0.0001) (Figure 2D). We confirmed these data by immunofluorescence microscopy, detecting a strong green signal in DENV-2 THP-1 Mø samples consistent with TF presence (Figure 2E). The increased levels of TF suggest that, during DENV infection, activated intermediate Mø represent a source of circulating TF, thus contributing to coagulation activation and thrombus formation [32].

Considering the procoagulant phenotype observed in monocytes in response to DENV infection, characterized by the expansion of activated intermediate monocytes and increased TF expression, we evaluated whether this activation state is associated with a proinflammatory response by measuring the mRNA expression of TNF-α and IL-8. Therefore, we analyzed lysates from Control THP-1 Mø, LPS-stimulated THP-1 Mø (LPS THP-1 Mø; positive control), and DENV-2 THP-1 Mø to assess proinflammatory cytokine expression at the mRNA level by RT–PCR amplification. Distinct TNF-α and IL-8 amplicon bands in LPS THP-1 Mø and DENV-2 THP-1 Mø samples were observed, but not in Control THP-1 Mø, supporting the transcriptional activation of proinflammatory cytokines (Figure 2F). Although the secretion of TNF-α and IL-8 proteins was not quantified, representing a limitation of the present study, the transcriptional detection of both cytokines provides initial molecular evidence that DENV infection induces a proinflammatory response in activated intermediate THP-1 monocytes. These findings support the activation of inflammatory pathways associated with the phenotypic changes observed following DENV infection.

These findings indicate that DENV infection drives a coordinated reprogramming of THP-1 Mø toward an activated intermediate phenotype with enhanced adhesion capacity and procoagulant and proinflammatory capabilities, providing a mechanistic framework to investigate their role in the generation of extracellular vesicles, particularly microparticles, and their potential contribution to vascular dysfunction described in SD.

### 2.2. DENV-Infected THP-1 Monocytes Release Microparticles Carrying Viral Proteins E and NS1 That May Contribute to Viral Dissemination to Endothelial Vascular Cells

**Figure 3:**
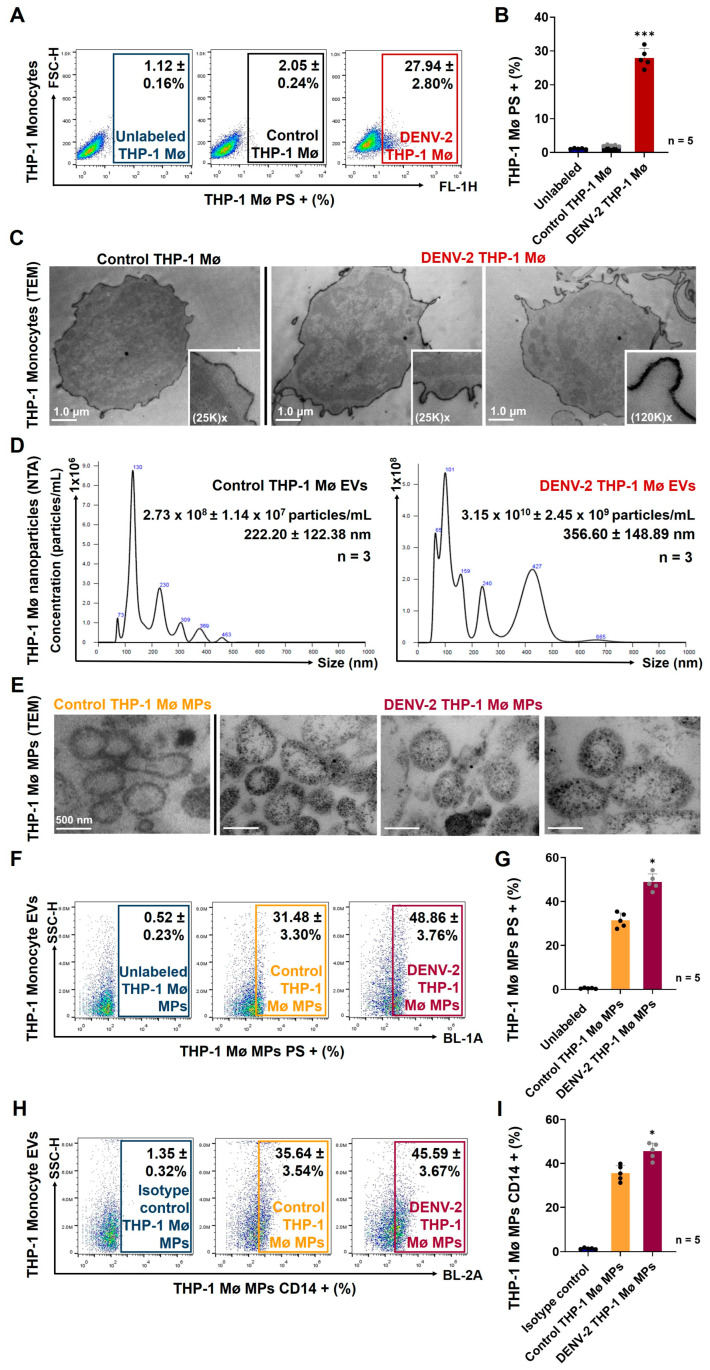
Characterization of EVs (MPs) released by THP-1 monocytes. (**A**) Detection of PS by Annexin V binding assay at 72 h p.i. (representative dot plots by FACS). (**B**) Percentages of PS-positive THP-1 Mø. The percentage of cells PS+ in DENV-2 THP-1 Mø was compared with Control THP-1 Mø using an unpaired Student’s *t*-test. Statistical significance is denoted as *** when *p* < 0.0001. (**C**) Transmission electron microscopy (TEM) images of uninfected (Control THP-1 Mø) and DENV-infected THP-1 Mø (DENV-2 THP-1 Mø) stained with ruthenium red (scale bar: 1.0 µm). (**D**) Nanoparticle tracking analysis (NTA) of THP-1 Mø-derived EVs (THP-1 Mø EVs). Representative histograms showing mean ± standard deviation of particle concentration (particles/mL) and size distribution. (**E**) TEM images of MPs isolated from Control THP-1 Mø and DENV-2 THP-1 Mø (scale bar: 500 nm). (**F**) Detection of PS by Annexin V binding assay in THP-1 Mø MPs isolates at 72 h p.i. (representative dot plots by FACS). (**G**) Percentages of PS-positive MPs. (**H**) Detection of CD14 in THP-1 Mø MPs isolates at 72 h p.i. (representative dot plots by FACS). (**I**) Percentages of CD14-positive MPs. The percentages of MPs PS+ and CD14+ from DENV-2 THP-1 Mø MPs were compared with Control THP-1 Mø MPs using an unpaired Student’s *t*-test. Statistical significance is denoted as * when *p* < 0.05 and *** when *p* < 0.001. Isotype control or unlabeled THP-1 Mø (blue), Control THP-1 Mø (black), DENV-2 THP-1 Mø (red), Control THP-1 Mø MPs (orange), and DENV-2 THP-1 Mø MPs (cherry). n = 3 or 5 independent experiments.

Microparticles (MPs) are a subset of EVs released from activated cells through membrane budding, resulting from calcium-mediated disruption of cytoskeletal anchorage and loss of phospholipid asymmetry, whereby phosphatidylserine (PS) becomes externalized from the inner to the outer membrane leaflet, allowing MPs release with PS exposed on the external surface [33,34]. Considering the differentiation and activation of DENV-infected THP-1 Mø in our experimental model, we evaluated the production of EVs (MPs) to determine their possible function in DENV-associated vasculopathy (Figure 3).

We evaluated PS exposure in the THP-1 monocyte plasma membrane to determine whether DENV-2 THP-1 Mø could release MPs at 72 h p.i. and found that 25% of DENV-2 THP-1 Mø were PS+ (*p* < 0.0001), in contrast to Control THP-1 Mø, where 2% of cells were PS+ (Figure 3A,B). Given that PS externalization at the plasma membrane can also be associated with dead cells, we assessed viable cell morphology by transmission electron microscopy (TEM) using ruthenium red staining, observing that DENV-2 THP-1 Mø exhibited abundant membrane protrusions suggestive of active budding, consistent with MPs release, whereas this was less evident in Control THP-1 Mø (Figure 3C). These findings indicate that MPs may be generated related to our experimental conditions and can be isolated for further characterization.

We adapted previously described protocols for EVs isolation by differential ultracentrifugation from 1.0 × 10^7^ THP-1 monocytes (see Section 4.9, Materials and Methods). The EV fractions corresponding to MPs pellets were characterized by nanoparticle tracking analysis (NTA) to determine particle concentration and size distribution. We found that DENV-2 THP-1 Mø released approximately 3.15 × 10^10^ particles/mL, with a mean size of 357 nm, representing 1.2- and 1.6-fold increases in concentration and size, respectively, compared with Control THP-1 Mø samples (Figure 3D). Morphologically, TEM images showed heterogenous populations of large EVs with well-defined lipid bilayers and a size consistent with MPs. In DENV-2 THP-1 Mø-derived MPs (DENV-2 THP-1 Mø MPs), we observed irregular and electron-dense internal structures, which were not observed in Control THP-1 Mø-derived MPs (Control THP-1 Mø MPs) (Figure 3E).

The monocyte membrane origin of EVs was evaluated in MPs isolates by detecting PS and CD14 at the surface membrane level by FACS. For DENV-2 THP-1 Mø MPs isolates, 49% were PS+ and 46% were CD14+, representing 1.6- (*p* < 0.05) and 1.3-fold (*p* < 0.05) increases in the levels of PS and CD14, respectively, compared with Control THP-1 Mø MPs (Figure 3F–I). These data show that in the EV fractions isolated from THP-1 monocytes, MPs are present.

**Figure 4:**
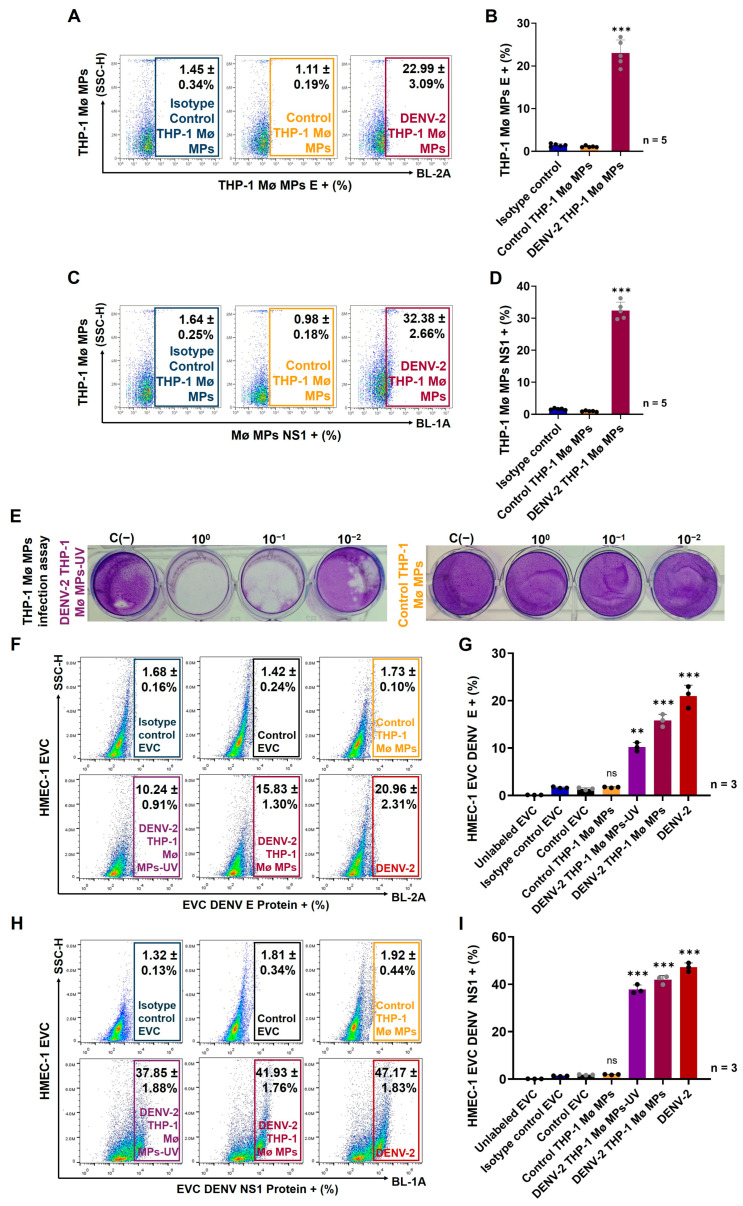
DENV-infected intermediate THP-1 Mø-derived MPs carry viral proteins E and NS1 and promote their transfer to EVC. (**A**) Detection of DENV E protein in THP-1 Mø MPs isolates at 72 h p.i. (representative dot plots by FACS). (**B**) Percentage of E-positive MPs. (**C**) Detection of DENV NS1 protein in THP-1 Mø MPs isolates at 72 h p.i. (representative dot plots by FACS). (**D**) Percentage of NS1-positive MPs. The percentages of E+ and NS1+ MPs from DENV-2 THP-1 Mø MPs isolates were compared with Control THP-1 Mø MPs isolates using an unpaired Student’s *t*-test. (**E**) Representative images of lytic plaque assays performed on Vero cells stimulated with DENV-2 THP-1 Mø-derived MPs-UV and Control THP-1 Mø-derived MPs. (**F**) Detection of DENV E protein in EVC stimulated with THP-1 Mø MPs isolates at 72 h post-stimulation. (**G**) Percentage of E+ EVC. (**H**) Detection of DENV NS1 protein in EVC stimulated with THP-1 Mø MPs isolates at 72 h post-stimulation. (**I**) Percentage of NS1+ EVC. The percentages of E+ and NS1+ EVC under different THP-1 Mø MPs stimuli were compared with Control EVC (uninfected cells) using a one-way ANOVA test. Statistical significance is denoted as ** when *p* < 0.01 and *** when *p* < 0.0001; ns = not significant. Unlabeled cells (light gray), Isotype control (blue), Control EVC (black), Control THP-1 Mø MPs (orange), DENV-2 THP-1 Mø MPs-UV (purple), DENV-2 THP-1 Mø MPs (cherry), and DENV-2 (red). n = 3 or 5 independent experiments.

The present results demonstrate that DENV-infected intermediate THP-1 monocytes actively release MPs, as evidenced by increased PS externalization, enhanced membrane budding, and the recovery of EVs fractions with size and morphology consistent with MPs. These EVs exhibit characteristic surface markers, including PS and CD14, confirming their monocyte membrane origin because of infection-induced activation. Notably, DENV infection significantly increased both the production and structural complexity of MPs, suggesting that these vesicles may serve as biological carriers in the context of infection. Therefore, we evaluated whether DENV-2 THP-1 Mø-derived MPs transport the viral proteins E and NS1, and whether exposure of endothelial vascular cells (EVC) to these vesicles in vitro may facilitate the transfer of these viral components (Figure 4).

We found that 23% of DENV-2 THP-1 Mø-derived MPs presented viral E protein at the membrane surface level (*p* < 0.0001) (Figure 4A,B). DENV-infected THP-1 Mø-derived MPs may acquire the viral E protein during membrane budding concomitant with virion release, allowing them to carry this viral material within their internal content, or through virus–MPs membrane interactions in the extracellular space [35,36,37,38]. This finding suggests two possible explanations. First, the E protein detected in MPs may originate from complete viral particles associated with these vesicles, potentially facilitating the transfer of viral material to recipient cells. Alternatively, the E protein may derive from infected cellular membranes incorporated during MPs biogenesis, where it could contribute to the modulation of host immune responses.

Importantly, we found that 32% of DENV-2 THP-1 Mø-derived MPs showed viral NS1 protein at the membrane surface (*p* < 0.0001) (Figure 4C,D). DENV NS1 protein is a key virulence factor implicated in vascular dysfunction through different mechanisms like glycocalyx degradation, disruption of intercellular junctions, and induction of acute inflammatory response that ultimately compromise vascular barrier integrity [39]. It has been reported that NS1 remains in a dimeric form in DENV-infected cells and is released in a multimeric form to the extracellular space [40], which could be acquired by EVs like exosomes [41]. This finding is particularly relevant in the context of DENV-associated vasculopathy, as MPs also may function as circulating carriers of NS1, enabling its delivery to EVC and plausibly amplifying vascular activation and tissue damage.

Together, our results indicate that DENV-infected THP-1 Mø-derived MPs carry viral E and NS1 proteins, supporting their potential role as carriers of pathogenic viral components and sustain a mechanism basis to evaluate their capacity to mediate viral dissemination and to induce functional alterations in target cells.

To determine the capacity of DENV-infected THP-1 Mø-derived MPs to mediate viral dissemination, we adapted a lytic plaque assay protocol (see Section 4.3, Materials and Methods) to evaluate plaque formation in confluent Vero cell monolayers following stimulation with MPs isolates. MPs-containing samples were obtained through a low-speed centrifugation step (16,500× *g* for 35 min at 4 °C) during the EVs isolation procedure. Under these conditions, large vesicles such as MPs are pelleted, whereas DENV virions are expected to remain in the supernatant due to their smaller size and lower sedimentation properties.

To further reduce potential contamination by free virions or viral RNA that may co-precipitate during MPs isolation and interfere with downstream functional assays, DENV-2 THP-1 Mø-derived MPs samples were washed twice with 1× PBS following UV inactivation at 1200 µJ (×100) for three consecutive cycles (see Section 4.13, Materials and Methods). This procedure was initially validated using DENV-2 viral stock, and the reduction of viral infectivity was confirmed by lytic plaque assay, detection of viral E protein in THP-1 Mø exposed to UV-inactivated samples (DENV-2 [UV] THP-1 Mø), and DENV-2 RNA detection by RT-PCR in THP-1 Mø exposed to UV-inactivated DENV-2 (Figure S1). Collectively, these controls support a substantial reduction in free infectious virions. In this context, following UV treatment of THP-1 Mø-derived MPs and subsequent PBS washes as described above, any residual infectivity observed is likely enriched in MPs-associated viral components, although the contribution of residual free virions cannot be completely excluded.

We observed that stimulation with UV-inactivated DENV-infected Mø-derived MPs (DENV-2 Mø MPs-UV) induced the formation of lytic plaques, indicating the presence of infectious viral components associated with these vesicles, whereas no plaques were detected following stimulation with Control Mø-derived MPs (Figure 4E). This finding suggests that DENV-2 THP-1 Mø-derived MPs may act as protective carriers of viral components, shielding them from UV-induced damage and preserving their activity.

We previously reported [42] that DENV infection of EVC directly promotes vasculopathy through active viral replication, leading to high levels of viremia and the activation of intracellular signaling pathways that favor vascular activation, tissue damage, and increased endothelial barrier. However, the transfer of viral components to naïve cells may represent an additional pathogenic mechanism capable of enhancing host immune responses and contributing to endothelial activation and damage. Therefore, we performed stimulation assays using THP-1 Mø MPs isolates on naïve EVC to evaluate their potential contribution to viral dissemination by assessing the presence of E and NS1 proteins on the endothelial cell surface.

We found that 10% of HMEC-1 EVC incubated with DENV-2 THP-1 Mø MPs-UV were E+ (*p* < 0.01), whereas 16% of EVC stimulated with DENV-2 THP-1 Mø MPs and 21% of EVC infected with DENV-2 (MOI 1) were E+ (*p* < 0.0001). Viral E protein was not detected in Control EVC (1%) or in cells stimulated with Control THP-1 Mø MPs (2%) (Figure 4F,G), suggesting that MPs-associated viral components containing E protein can be transferred to naïve EVC, promoting viral dissemination.

In parallel, 38% of HMEC-1 EVC stimulated with DENV-2 THP-1 Mø MPs-UV were NS1+ (*p* < 0.0001), whereas 42% of HMEC-1 EVC stimulated with DENV-2 THP-1 Mø MPs and 47% of HMEC-1 EVC infected with DENV-2 (MOI 1) were NS1+ (*p* < 0.0001). NS1 protein was not detected in Control EVC (2%) or in Control THP-1 Mø MPs-stimulated cells (2%) (Figure 4H,I). The presence of NS1 indicates that EVC may be susceptible to NS1-mediated activation, injury, and inflammatory processes associated with vascular dysfunction.

These data support that DENV-2-infected THP-1 Mø-derived MPs facilitate viral dissemination by the interaction with naïve EVC. The effect observed in HMEC-1 EVC stimulated with DENV-2 THP-1 Mø-derived MPs (16% E+ EVC and 42% NS1+ EVC) may represent the experimental condition that most closely resembles the in vivo scenario, where both MPs biogenesis and the DENV replicative cycle occur simultaneously in infected monocytes, and recipient endothelial cells may be exposed to both MPs-associated viral proteins (E and NS1) and infectious DENV particles. In contrast, HMEC-1 EVC stimulated with UV-treated DENV-2 THP-1 Mø-derived MPs (10% E-positive EVC and 36% NS1-positive EVC) exhibited a reduced contribution from free infectious virions. These findings suggest that MPs contain viral proteins and may protect them from physical damage, such as UV irradiation, thereby facilitating their transfer to naïve cells.

Taken together, our results suggest that DENV infection induces a functional reprogramming of THP-1 monocytes that promotes the release of MPs carrying viral proteins (E and NS1). UV-treated DENV-2 THP-1 Mø MPs were associated with the presence of infectious viral components, as evidenced by lytic plaque formation in Vero cells, suggesting that viral components may be protected within or associated with these vesicles. Furthermore, MPs derived from DENV-2-infected THP-1 Mø facilitated the transfer of viral proteins to EVC, supporting their potential contribution to viral dissemination. In parallel, the presence of NS1 highlights a possible mechanism through which these vesicles may contribute to endothelial activation, vascular dysfunction, and damage. Together, these findings support a model in which THP-1 monocyte-derived MPs act as Trojan-like vehicles for viral components, potentially contributing to viral dissemination and endothelial damage associated with SD.

### 2.3. DENV-Infected THP-1 Mø-Derived MPs Exhibit Procoagulant Activity and Promote Vascular Dysfunction and Endothelial Vascular Barrier Disruption

Dengue-associated vasculopathy is characterized by functional and structural alterations in the vascular endothelium following DENV infection, leading to a shift toward a procoagulant, proinflammatory, and proadherent phenotype in EVC, ultimately resulting in EVC damage, increased permeability, and plasma leakage [4,43]. Vasculopathy, as a pathognomonic sign of SD, results from the convergence of multiple factors, including viral serotype, high viral load, antibody-dependent enhancement, platelet activation, and the release of proinflammatory mediators; however, its underlying mechanisms remain poorly understood [44,45].

**Figure 5:**
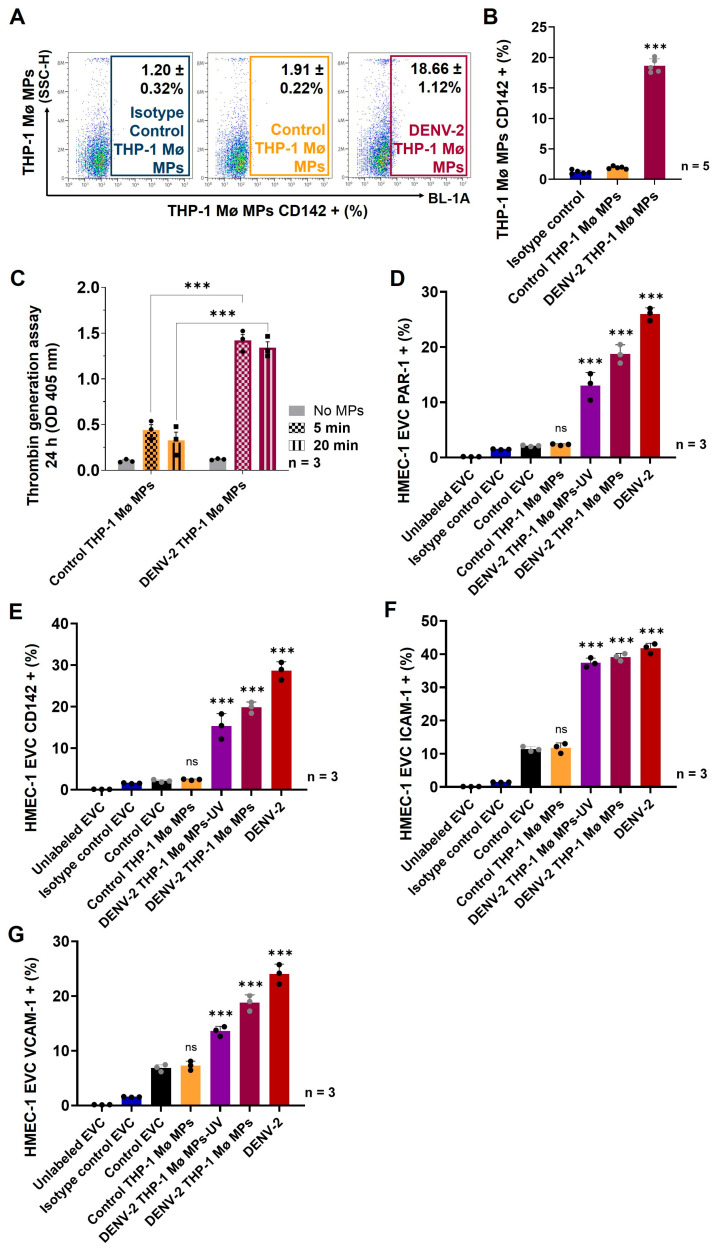
Procoagulant DENV-2 THP-1 Mø-derived MPs promote a shift toward a procoagulant, proinflammatory, and proadherent phenotype in EVC. (**A**) Detection of TF (CD142) in Mø THP-1 MPs isolates at 72 h p.i. (representative dot plots by FACS). (**B**) Percentage of TF+ MPs. The percentage of TF+ MPs from DENV-2 THP-1 Mø MPs isolates was compared with Control THP-1 Mø MPs isolates using an unpaired Student’s *t*-test. (**C**) Thrombogenicity assay using THP-1 Mø MPs isolates at 24 h post-incubation. Thrombin generation time (5 and 20 min) was analyzed using a two-way ANOVA test. (**D**) Percentage of PAR-1+ HMEC-1 EVC stimulated with THP-1 Mø MPs isolates at 72 h post-stimulation. (**E**) Percentage of TF+ HMEC-1 EVC stimulated with THP-1 Mø MPs isolates at 72 h post-stimulation. (**F**) Percentage of ICAM-1 (CD54)+ HMEC-1 EVC stimulated with THP-1 Mø MPs isolates at 72 h post-stimulation. (**G**) Percentage of VCAM-1 (CD106)+ HMEC-1 EVC stimulated with THP-1 Mø MPs isolates at 72 h post-stimulation. The percentages of PAR-1, TF+, ICAM-1+, and VCAM-1+ HMEC-1 EVC under different THP-1 Mø MPs stimuli were compared with Control EVC (uninfected cells) using a one-way ANOVA test. Statistical significance is denoted as *** when *p* < 0.0001; ns = not significant. Unlabeled cells (light gray), Isotype control (blue), Control EVC (black), Control THP-1 Mø MPs (orange), DENV-2 THP-1 Mø MPs-UV (purple), DENV-2 THP-1 Mø MPs (cherry), and DENV-2 (red). n = 3 or 5 independent experiments.

Our data show that DENV-2-infected THP-1 Mø-derived MPs carry E and NS1 viral proteins and promote viral dissemination to EVC, potentially predisposing them to damage through the accumulation of NS1 at the cell surface. Considering that these MPs originate from activated intermediate THP-1 Mø with a TF+ phenotype and are enriched in PS, we evaluated their procoagulant potential by assessing TF presence at their membrane surface and their capacity to generate thrombin, as well as their contribution to vascular endothelium dysfunction (Figure 5).

We found that 19% of DENV-2 THP-1 Mø-derived MPs were TF+ (*p* < 0.0001), whereas 2% of Control Mø-derived MPs were TF+, representing a 9.5-fold increase (Figure 5A,B). This finding suggests that DENV-2 THP-1 Mø-derived MPs may act as a source of circulating TF and anionic phospholipids (PS) (Figure 3F,G), thereby contributing to activation of the coagulation cascade.

To assess their functional procoagulant activity, we evaluated the thrombin generation capacity of DENV-2 THP-1 Mø-derived MPs using a colorimetric assay based on thrombin enzymatic activity on a chromogenic substrate at different time points (Figure 5C; see Section 4.15, Materials and Methods). We found that DENV-2 THP-1 Mø-derived MPs promoted thrombin generation by approximately 3.0-fold more, compared with Control THP-1 Mø-derived MPs at 24 h post-incubation, using platelet-poor plasma as a source of coagulation factors. The peak enzymatic activity was detected at 5 min after substrate addition (*p* < 0.0001), supporting their capacity to activate the coagulation cascade and promote a prothrombotic state.

These results indicate that DENV-2 THP-1 Mø-derived MPs contain functional procoagulant elements (PS+ TF+), which may contribute to the establishment of a prothrombotic activity during progression to coagulopathy and potentially as an active actor in the development of disseminated intravascular coagulation (DIC) as present in SD cases [43].

Based on our previous findings that DENV-2 THP-1 Mø-derived MPs carry viral components and promote viral dissemination to EVC, underlining that these MPs transport NS1, a key virulence factor known to induce vascular dysfunction. Together with their procoagulant capacity, these MPs may enhance thrombin generation at the endothelial interface. This convergence of viral and coagulation-dependent mechanisms suggests that DENV-2 THP-1 Mø-derived MPs act as multifunctional effectors of vascular activation and damage.

Given their capacity to generate thrombin, DENV-2 THP-1 Mø-derived MPs may directly influence EVC activation. Thrombin is a key effector of the coagulation cascade that activates EVC through protease-activated receptor (PAR-1) signaling, promoting inflammatory responses, expression of adhesion molecules, and a procoagulant phenotype [42,46]. Therefore, we assessed whether stimulation of HMEC-1 EVC with DENV-2 THP-1 Mø-derived MPs induces PAR-1, TF, and proadherent molecules (intercellular adhesion molecule type 1 [ICAM-1] and vascular cell adhesion molecule type 1 [VCAM-1]) expression, to establish a mechanistic link between MPs-induced thrombin generation and vascular dysfunction characteristic of DENV-associated vasculopathy.

In a procoagulant and prothrombotic environment, the presence and/or upregulation of PAR-1 in EVC reflects their ability to sense and respond to thrombin and other coagulation proteases, thereby mediating the crosstalk between coagulation and inflammation, including the induction of proinflammatory cytokine signaling [42,47].

In response to stimulation with DENV-2 THP-1 Mø MPs-UV, 13% of HMEC-1 EVC were PAR-1+, representing an approximately 6.2-fold increase (*p* < 0.0001) compared with cells stimulated with Control EVC (2.1%, representing cells in a basal state) and Control THP-1 Mø MPs (2.3%). PAR-1 levels further increased in HMEC-1 EVC stimulated with DENV-2 THP-1 Mø MPs (19%) and in DENV-2-infected HMEC-1 EVC (26%) (*p* < 0.0001) (Figure 5D). This data supports that DENV-2 THP-1 Mø MPs may have a role as mediators of thrombin-responsive signaling pathways. Although Control THP-1 Mø-derived MPs expressed low levels of TF that may support thrombin generation, PAR-1 levels in stimulated cells were not significantly different and remained comparable to those observed in Control EVC. This upregulation of PAR-1 suggests that procoagulant DENV-2 THP-1 Mø-derived MPs, including those subjected to UV treatment, may increase the responsiveness of HMEC-1 EVC to coagulation proteases, potentially contributing to proinflammatory and procoagulant processes associated with vascular activation and dysfunction.

Consistent with the activation of thrombin-responsive signaling pathways mediated by PAR-1, we next evaluated whether this response translates into a procoagulant vascular endothelium phenotype, as shown by increased TF levels. We found that 15% of HMEC-1 EVC stimulated with DENV-2 THP-1 Mø MPs-UV were TF+, representing an approximately 7.2-fold increase (*p* < 0.0001) compared with Control EVC (2.1%) and Control THP-1 Mø MPs-stimulated EVC (2.4%). As controls, 20% of HMEC-1 EVC stimulated with DENV-2 THP-1 Mø MPs and 29% of DENV-2-infected HMEC-1 EVC were TF+, respectively (*p* < 0.0001) (Figure 5E). The increased expression of TF observed in HMEC-1 EVC stimulated with DENV-2 THP-1 Mø MPs suggests that these vesicles may contribute to the acquisition of procoagulant endothelium phenotype. In contrast, stimulation with Control THP-1 Mø-derived MPs did not induce significant changes in TF expression, remaining comparable to basal endothelial levels (Control EVC). This observation is consistent with the role of physiological Mø-derived MPs in maintaining vascular homeostasis and supports the notion that DENV infection alters the biological properties of MPs toward a phenotype associated with coagulation activation and vascular dysfunction.

Our results indicate that DENV-2 THP-1 Mø MPs induce procoagulant and proinflammatory phenotypes in HMEC-1 EVC characterized by increased PAR-1 and TF levels. This response suggests that MP-driven thrombin generation not only enhances vascular endothelium sensitivity to coagulation proteases but also promotes a sustained activation state that may amplify local coagulation, inflammation, and EVC dysfunction. Notably, the effect observed following stimulation with UV-treated DENV-2 THP-1 Mø-derived MPs indicates that THP-1 Mø MPs-associated viral components may participate in endothelial PAR-1 and TF upregulation independently of free infectious virions.

As this activated phenotype is closely linked to vascular inflammation and leukocyte adhesion, we evaluate if DENV-2 THP-1 Mø-derived MPs promote the presence of the proadherent molecules ICAM-1 and VCAM-1 in HMEC-1 EVC.

We found that 37% and 14% of HMEC-1 EVC stimulated with DENV-2 THP-1 Mø MPs-UV were ICAM-1+ and VCAM-1+, respectively, representing approximately a 3.3-fold increase in ICAM-1 expression and a 2.0-fold increase in VCAM-1 expression (*p* < 0.0001) compared with Control EVC, which expressed basal levels for these markers (11% ICAM-1+ and 7% VCAM-1+) and Control Mø MPs-stimulated EVC (12% ICAM-1+ and 7% VCAM-1+) that did not show significant differences compared Control EVC. In HMEC-1 EVC stimulated with DENV-2 THP-1 Mø MPs, 39% of cells were ICAM-1+ and 19% were VCAM-1+, whereas in DENV-2-infected HMEC-1 EVC, 42% and 24% of cells were ICAM-1+ and VCAM-1+, respectively (Figure 5F,G). These results indicate that DENV-2-infected THP-1 Mø-derived MPs promote endothelium activation toward a proinflammatory and proadherent phenotype, favoring leukocyte adhesion (e.g., intermediate monocytes) and suggesting a compromised EVC barrier that may contribute to vascular leakage.

**Figure 6:**
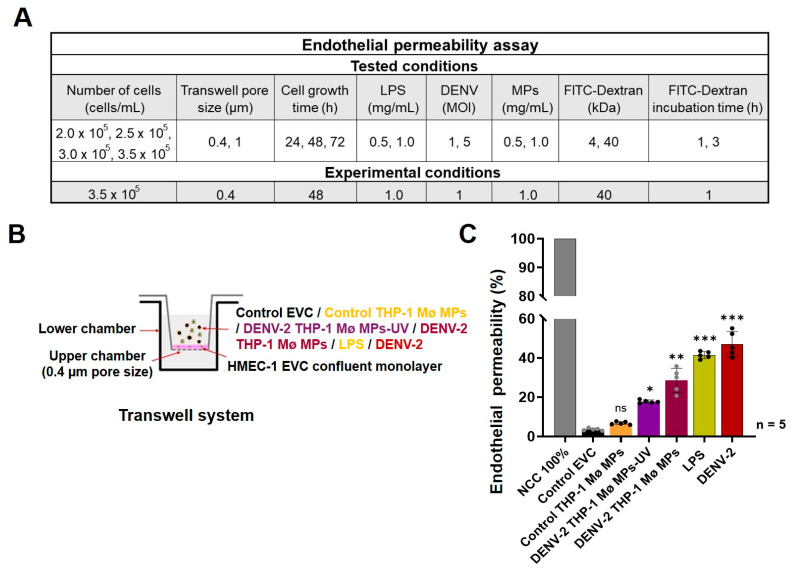
DENV-2-infected THP-1 Mø-derived MPs increase vascular permeability in HMEC-1 EVC. (**A**) Overview of the tested conditions and the experimental parameters established for the HMEC-1 EVC permeability assay. (**B**) Schematic representation of the Transwell system used for the HMEC-1 EVC permeability assay. (**C**) Endothelial permeability percentages were determined by measuring fluorescein isothiocyanate (FITC)-dextran that crossed HMEC-1 EVC monolayers following stimulation with THP-1 Mø MPs isolates. For 100% permeability, a FITC-dextran–only control (no-cell control, NCC) was used. Permeability percentages were compared with Control EVC using a one-way ANOVA test. Statistical significance is denoted as * when *p* < 0.05, ** when *p* < 0.01, and *** when *p* < 0.0001; ns = not significant. NCC 100% (dark gray), Control EVC (black), Control THP-1 Mø MPs (orange), DENV-2 THP-1 Mø MPs-UV (purple), DENV-2 THP-1 Mø MPs (cherry), LPS (dark yellow), and DENV-2 (red). n = 5 independent experiments.

We have demonstrated that DENV-2 THP-1 Mø-derived MPs (PS+ NS1+ TF+) promote vascular endothelium activation characterized by procoagulant, proinflammatory, and proadherent responses, features consistent with DENV-associated vasculopathy. Therefore, we evaluated whether stimulation with these THP-1 Mø MPs compromises endothelial barrier integrity. Increased endothelial vascular permeability is a hallmark of SD and reflects disruption of endothelial junctions and alteration of vascular barrier function [43]. In this context, we assessed whether exposure to DENV-2 THP-1 Mø-derived MPs induces changes consistent with vascular barrier dysfunction using Transwell assays (Figure 6).

First, we established the optimal conditions for the permeability assays (Figure 6A,B), in which fluorescence from FITC-dextran crossing the Transwell membrane was measured to calculate endothelial vascular cells’ permeability percentages (p%). We found that stimulation with DENV-2 THP-1 Mø-derived MPs increased EVC permeability. The p% values of 4% in Control EVC, 7% in HMEC-1 EVC stimulated with Control THP-1 Mø MPs, 18% in HMEC-1 EVC stimulated with DENV-2 THP-1 Mø MPs-UV (*p* < 0.05), 29% in HMEC-1 EVC stimulated with DENV-2 THP-1 Mø MPs (*p* < 0.01), 42% in LPS-stimulated HMEC-1 EVC, and 47% in DENV-infected HMEC-1 EVC (*p* < 0.001) were obtained (Figure 6C). The permeability induced by stimulation with DENV-2 THP-1 Mø MPs-UV represented a 4.5- and 2.6-fold increase compared with Control EVC and HMEC-1 EVC-stimulated with Control THP-1 Mø MPs, respectively.

We observed a 1.6-fold reduction (*p* < 0.05) in HMEC-1 EVC barrier permeability following stimulation with DENV-2 THP-1 Mø-derived MPs-UV compared with permeability induced by DENV-2 THP-1 Mø-derived MPs. This finding suggests that, although UV treatment reduces the potential contribution of free infectious virions, MPs-associated cellular (PS and TF) and viral components retain the ability to compromise endothelial barrier integrity. Furthermore, the persistence of a measurable biological effect after UV irradiation supports the possibility that MPs-associated cellular and viral proteins, including NS1, may contribute to endothelial activation and vascular dysfunction.

These data demonstrate that DENV-2-infected THP-1 Mø-derived MPs promote endothelial barrier dysfunction, leading to increased vascular permeability, a key feature of DENV-associated vasculopathy.

The present data supports that following DENV-2 infection, THP-1 monocytes undergo sustained functional cell modifications that promote the release of microparticles, carrying viral components and procoagulant determinants. The DENV-infected THP-1 Mø-derived MPs (PS+, TF+, E+/NS1+) act as multifunctional effectors that favor viral dissemination with coagulation and inflammatory responses. Functionally, these vesicles promote thrombin generation and HMEC-1 EVC activation, inducing a procoagulant, proinflammatory, and proadherent phenotype characterized by increased levels of PAR-1, TF, ICAM-1, and VCAM-1. This activated state ultimately compromises endothelial barrier integrity, leading to increased vascular barrier permeability, a hallmark of DENV-associated vasculopathy. Together, these results support a mechanistic model in which monocyte-derived MPs contribute to the amplification of vascular dysfunction during DENV infection, highlighting their relevant role in the pathogenesis of SD.

## 3. Discussion

The incidence of dengue fever has increased significantly worldwide in recent decades. About half of the world’s population is at risk of DENV infection [1,3]. The main impacts of disease are on human health and national and global economies. Severe dengue is a major cause of morbidity and mortality, particularly in Asia and Latin America. Currently, no safe vaccine or specific treatment for DENV infection are available. The pathogenesis of SD is characterized by high viremia levels (low rate of viral clearance), increased EVC activation, enhanced production of proinflammatory mediators, increased vascular permeability, plasma extravasation, and EVC injury. The vascular endothelium plays a determining role in the response to injury because it functions as a regulatory interface during hemostasis (coagulation–fibrinolysis–inflammation) and as a key regulator of endothelial barrier integrity [48]. During DENV infection, EVC, monocytes, and immune cells are among the major cellular targets affected through direct and indirect mechanisms. In this context, EVC dysfunction is strongly associated with adverse clinical outcomes [44,49].

We previously reported that DENV infection of human EVC upregulates TF expression, triggering the generation of hemostatic proteases such as thrombin, which in turn activate PAR-1 and downstream inflammatory signaling pathways. These events promote the expression of adhesion molecules, including VCAM-1, and proinflammatory mediators such as IL-8, supporting their role in dengue-associated vasculopathy [42]. However, it remains unclear whether microparticles derived from viral target cells like monocytes, as product of cell activation in response to infection, may contribute to the pathogenic mechanisms associated with SD, including dysregulation of coagulation and inflammation process, increased vascular permeability with plasma extravasation, and endothelial dysfunction which may progress to shock, multiorgan failure, and death [37,50,51].

**Figure 7:**
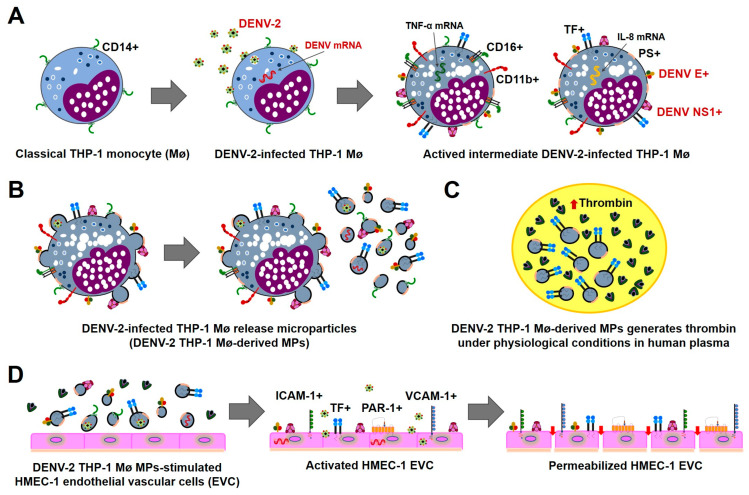
Microparticles from DENV-infected THP-1 monocytes mediate EVC activation and promote procoagulant, proinflammatory, and proadherent responses leading to increased barrier permeability (Graphical overview). (**A**) DENV infection induces functional reprogramming of monocytes toward an activated intermediate phenotype with proinflammatory (at transcriptional level) and procoagulant features. (**B**) Activated intermediate DENV-2-infected THP-1 monocytes release microparticles CD14+, PS+, and TF+ carrying viral E and NS1 proteins. (**C**) Microparticles derived from DENV-2-infected THP-1 monocytes exhibit a procoagulant profile (TF+/PS+) that promotes thrombin generation. (**D**) Upon interaction with HMEC-1 EVC, microparticles derived from DENV-2-infected THP-1 monocytes favor viral dissemination, activation, and induce a shift toward a procoagulant, proinflammatory, and proadherent phenotype, ultimately compromising barrier integrity and leading to increased vascular permeability.

Our findings demonstrate that DENV-2 infection induces a functional reprogramming of THP-1 monocytes toward an activated intermediate phenotype with proinflammatory (demonstrated here at the transcriptional level) and procoagulant characteristics, leading to the release of EVs, predominantly MPs. These DENV-2-infected THP-1 Mø-derived MPs may act as multifunctional effectors that integrate viral dissemination with coagulation and inflammatory responses. We found that these vesicles transport viral proteins (E+/NS1+) capable of facilitating the transfer of viral components to EVC, while simultaneously exhibiting a procoagulant profile (PS+/TF+) capable of supporting thrombin generation and activation of thrombin-responsive signaling pathways in HMEC-1 EVC (Figure 7A–C).

To reduce potential contamination by free virions or viral RNA, preparations of DENV-2 THP-1 Mø-derived MPs were subjected to UV treatment followed by PBS washing steps. This procedure was validated using DENV-2 viral stock, where the reduction of viral infectivity was confirmed by plaque assay, E protein detection, and RT-PCR amplification (Figure S1). These controls support a substantial reduction in free infectious virions. Therefore, following UV treatment and washing procedures, the residual effects observed are likely enriched in MPs-associated viral components, although the contribution of residual free virions cannot be completely excluded. Importantly, the persistence of viral components and biological activity after UV treatment suggests that DENV-2 THP-1 Mø-derived MPs may act as protective carriers of viral components, shielding them from UV-induced damage and preserving their biological activity.

As mentioned above, we previously reported that DENV infection directly promotes endothelial dysfunction [42]. In the present study, the transfer of viral proteins to naïve HMEC-1 EVC following stimulation with DENV-2 THP-1 Mø-derived MPs E+/NS1+ suggests an additional pathogenic mechanism through which infected monocytes may contribute to disease progression. Although the presence of viral proteins on HMEC-1 EVC does not demonstrate productive infection, it supports the possibility that MPs-associated viral components contribute to viral dissemination and cell activation. The detection of NS1 in stimulated HMEC-1 EVC is particularly relevant because this viral protein has been implicated in endothelial activation, glycocalyx disruption, inflammatory responses, and vascular leakage [39,41]. Therefore, the presence of NS1 suggests that EVC may be susceptible to NS1-mediated activation, injury, and inflammatory processes associated with vascular dysfunction.

At the endothelial level, we found that DENV-2-infected THP-1 Mø-derived MPs stimulation induces a shift toward a procoagulant, proinflammatory, and proadherent phenotype, characterized by increased expression of TF, PAR-1, ICAM-1, and VCAM-1. These findings indicate the establishment of a sustained activation state that ultimately compromises endothelial barrier integrity, leading to increased vascular permeability, a hallmark of dengue-associated vasculopathy and a critical event in the progression to severe dengue (Figure 7D). Notably, HMEC-1 EVC-stimulated with UV-treated DENV-2 THP-1 Mø-derived MPs maintained increased expression of cellular markers. These findings suggest that MPs-associated viral components may contribute to endothelial activation independently of free infectious virions.

The observed reduction in endothelial permeability following stimulation with UV-treated DENV-2 THP-1 Mø-derived MPs compared with untreated MPs further supports this interpretation. Although UV treatment substantially reduced the potential contribution of free infectious virions, MPs-associated viral components retained the ability to compromise endothelial barrier integrity. The persistence of these effects after UV treatment supports the possibility that viral proteins associated with the MPs surface or incorporated within their cargo contribute to endothelial activation, vascular dysfunction, and increased permeability.

Taken together, our results suggest that DENV infection induces a functional reprogramming of THP-1 monocytes that promotes the release of MPs carrying viral E and NS1 proteins. UV-treated DENV-2 THP-1 Mø-derived MPs remained associated with infectious viral components, as evidenced by lytic plaque formation in Vero cells, suggesting that viral components may be protected within or associated with these vesicles. Furthermore, MPs derived from DENV-2-infected THP-1 monocytes facilitated the transfer of viral proteins to EVC, supporting their potential contribution to viral dissemination. These findings support a model in which DENV-2-infected THP-1 Mø MPs contribute to the amplification of vascular dysfunction following DENV infection, acting as intermediaries where converge viral dissemination with host-derived pathogenic responses.

From a translational perspective, these results highlight the potential of monocyte-derived MPs as both biomarkers of disease severity and targets for therapeutic intervention, particularly in the context of endothelial dysfunction and dysregulated coagulation. Further characterization of MPs-associated cargo and signaling pathways may provide new insights into host–virus interactions that underlie progression to the severe forms of dengue.

Although these findings were generated using in vitro models that may not fully recapitulate the complexity of the in vivo environment, they provide a controlled framework for dissecting cellular and molecular mechanisms that are difficult to isolate in human disease. Moreover, the lack of animal models that faithfully reproduce the full spectrum of SD pathogenesis in humans underscores the value of these experimental approaches for the elucidation of the mechanisms involved in the tissue injury process. In this context, our data may be interpreted as evidence of biologically plausible pathways during EVC DENV-mediated damage. Importantly, while DENV-infected THP-1 Mø MPs do not exceed DENV in their intrinsic infectivity, our findings support their role as complementary mediators that facilitate and may enhance viral dissemination and amplify host responses associated with vascular dysfunction. Therefore, microparticles derived from DENV-infected monocytes should be considered as relevant contributors to disease progression that warrant further investigation in more complex biological systems.

Likewise, this additional dissemination mechanism may contribute to understanding viral persistence and pathogenesis by facilitating the transfer of viral components to distal or less-permissive cells. EVs-mediated viral spread provides foundational insights into DENV virulence and identifies potential targets for therapeutic strategies aimed at disrupting viral dissemination and limiting disease progression.

## 4. Materials and Methods

### 4.1. Cell Cultures

Monkey kidney epithelial cells (Vero; ATCC CCL-81, USA) and human peripheral blood THP-1 monocytes (ATCC TIB-202) were cultured in Dulbecco’s Modified Eagle Medium (DMEM; Biowest, Riverside, MO, USA) and RPMI-1640 medium (Biowest), respectively. Media were supplemented with 10% (*v*/*v*) fetal bovine serum (FBS; Biowest), 2 mM L-glutamine (Biowest), and a 1× antibiotic–antimycotic solution containing 100 U/mL penicillin, 0.1 mg/mL streptomycin, and 0.25 μg/mL amphotericin B (Biological Industries, Cromwell, CT, USA).

Human microvascular endothelial cells (HMEC-1; ATCC CRL-3243) were maintained in MCDB 131 medium (Sigma-Aldrich, St. Louis, MO, USA) supplemented with 10% FBS, 2 mM L-glutamine, 1× antibiotic–antimycotic solution, 10 ng/mL epidermal growth factor (Sigma-Aldrich), and 1 μg/mL hydrocortisone (Sigma-Aldrich). All cell cultures were maintained at 37 °C in a humidified atmosphere containing 5% CO_2_.

### 4.2. Isolation, Propagation, and Purification of Dengue Virus

The prototype dengue virus serotype 2 strain New Guinea C (DENV-2 NGC) was used throughout the study. Viral stocks were propagated in confluent Vero cell monolayers at a MOI of 0.5 and maintained at 37 °C with 5% CO_2_ for 7 days. Culture supernatants were harvested and clarified by low-speed centrifugation (GH3.8 rotor, Beckman GPR Centrifuge; Beckman Coulter, Inc., Brea, CA, USA) at 200× *g* for 15 min at room temperature (rt) to remove cellular debris.

Clarified DENV-2 supernatants were concentrated by precipitation with 10% polyethylene glycol 8000 and 15% NaCl and further purified by ultracentrifugation through a discontinuous sucrose gradient (5–50%, *w*/*v*) using an SW28 rotor in a Beckman XL-90 ultracentrifuge (Beckman Coulter, Inc.) at 120,000× *g* for 2 h at 4 °C. Purified viral fractions were collected, aliquoted, and stored at −72 °C until use.

### 4.3. Titration of Dengue Virus

Confluent Vero cell monolayers grown in 12-well culture plates (Corning, Inc., Corning, NY, USA) were infected with 450 µL of 10-fold serial dilutions of the viral stock in duplicate and incubated for 2 h at 37 °C with 5% CO_2_. Then, viral inoculum was removed, and cells were overlaid with DMEM supplemented with 1% carboxymethylcellulose (Sigma-Aldrich) and 2.5% FBS. Cultures were incubated at 37 °C with 5% CO_2_ for 14 days. At the end of the incubation period, the media were removed, and the monolayers were fixed with 96% methanol (J.T. Baker, Estado de México, Mexico) and stained with 1% crystal violet (Sigma-Aldrich). Plaques were enumerated manually, and viral titers were calculated and expressed as plaque-forming units per milliliter (PFU/mL).

### 4.4. Preparation of Extracellular Vesicle-Depleted Fetal Bovine Serum (EV-Depleted FBS)

To minimize contamination from serum-derived EVs and ensure the purity of isolated EVs, FBS was depleted of EVs prior to use as follows: Samples were first clarified by centrifugation at 900× *g* for 10 min at 4 °C, followed by filtration through a 0.22 µm pore size membrane (Merck, Rahway, NJ, USA). Clarified serum was then ultracentrifuged at 120,000× *g* for 18 h at 4 °C. The EV-depleted supernatant was recovered, transferred to new sterile conical tubes, and stored at 2 °C until use.

### 4.5. THP-1 Monocyte DENV-Infection Assays

THP-1 monocytes (THP-1 Mø; 1.0 × 10^6^ cells) were seeded in 25 cm^2^ cell culture flasks (Corning), infected with DENV-2 at an MOI of 1 using non-supplemented RPMI-1640 medium, and incubated for 2 h at 37 °C with 5% CO_2_. Cells were harvested, washed with 1× phosphate-buffered saline (PBS), and centrifuged at 550× *g* (Eppendorf 5415 R centrifuge; Merck, Darmstadt, Germany) for 10 min at rt in duplicate. Cell pellets were resuspended in RPMI-1640 medium supplemented with 5% EV-depleted FBS and incubated for 72 h.

### 4.6. Detection of DENV Proteins and Cellular Markers in THP-1 Monocytes by Flow Cytometry

THP-1 Mø (1.0 × 10^6^ cells per condition) were harvested and centrifuged at 550× *g* for 10 min at 4 °C. Cell pellets were washed with PBS, fixed with 2% paraformaldehyde (PFA; Sigma-Aldrich) in PBS for 5 min at 4 °C, and blocked with 2% bovine serum albumin (BSA; Biowest) in PBS for 30 min at rt to minimize nonspecific antibody binding. Cells were resuspended in 0.5% BSA in PBS and maintained at 4 °C until use.

For direct immunostaining, THP-1 cells were incubated with fluorophore-conjugated antibodies diluted 1:100 in 0.5% BSA in PBS for 30 min at rt, protected from light. Between incubation steps, cells were washed with 0.5% BSA in PBS and centrifuged at 550× *g* for 10 min at 4 °C. The following antibodies were used: phycoerythrin (PE)-conjugated mouse anti-human CD14 IgG1 (clone HCD14; Cat. #325606; BioLegend, San Diego, CA, USA); PE-conjugated mouse anti-human CD11b IgG1 (clone ICRF44; Cat. #301306; BioLegend); and fluorescein isothiocyanate (FITC)-conjugated mouse anti-human CD142 (Cat. #13133-MM05-F; Sino Biological, Wayne, PA, USA).

For indirect immunostaining, THP-1 cells were incubated with primary antibodies diluted 1:300 in 0.5% BSA in PBS overnight at 4 °C, followed by incubation with the corresponding fluorophore-conjugated secondary antibodies diluted 1:500 in 0.5% BSA in PBS for 2 h at rt, protected from light. The primary antibodies used were mouse anti-dengue complex monoclonal antibody (clone D3-2H2-9-21; Cat. #MAB8705; Sigma-Aldrich); rabbit anti-dengue virus NS1 protein (Cat. #GTX124280; GeneTex, Irvine, CA, USA); and mouse anti-human CD16 IgG1 (Cat. #555404; BD Pharmingen, BD Biosciences, San Jose, CA, USA). The secondary antibodies employed were Alexa Fluor 555-conjugated donkey anti-mouse IgG (Cat. #A-31570; Thermo Fisher Scientific, Waltham, MA, USA) and Alexa Fluor 488-conjugated donkey anti-rabbit IgG (Cat. #711-546-152; Jackson ImmunoResearch, West Grove, PA, USA).

For PS detection, THP-1 cells were resuspended in 1× Annexin V binding buffer (Cat. #556454; BD Pharmingen) and incubated with FITC-conjugated Annexin V (Cat. #640906; BioLegend) diluted at 1:300 for 30 min at rt protected from light.

The following antibodies were used as isotype controls: PE-conjugated mouse IgG1 antibody (clone MOPC-21; Cat. #400112; BioLegend); FITC-conjugated mouse IgG1 antibody (clone MOPC-21; Cat. #400107; BioLegend); mouse IgG1 antibody (clone P3.6.2.8.1; Cat. #14-4714-82; eBioscience, San Diego, CA, USA); and rabbit polyclonal antibody (clone Poly29108; Cat #910801; BioLegend).

Flow cytometry data acquisition was performed using FACSCalibur (BD Biosciences, Franklin Lake, NJ, USA) and Attune (Applied Biosystems, Thermo Fisher Scientific, Waltham, MA, USA) flow cytometers. Data were collected using CellQuest software version 5.1 and analyzed with FlowJo software version 10.

### 4.7. Detection of DENV E Protein and Tissue Factor in THP-1 Monocytes by Immunofluorescence

THP-1 Mø (2.5 × 10^5^ cells per condition) were seeded onto 8-well chamber slides (Lab-Tek II; Thermo Fisher Scientific), fixed with 2% PFA for 5 min at 4 °C, and blocked with 2% BSA in PBS for 30 min at rt.

For DENV E protein detection, THP-1 cells were incubated with a mouse anti-dengue complex antibody diluted 1:300 in 0.5% BSA in PBS overnight at 4 °C, followed by incubation with an Alexa Fluor 555–conjugated secondary antibody (1:500) for 2 h at rt, protected from light. The mouse IgG1 antibody clone P3.6.2.8.1 was used as an isotype control.

For Tissue Factor (CD142) detection, cells were incubated with a FITC-conjugated mouse anti-CD142 diluted 1:100 in 0.5% BSA in PBS for 2 h at rt, protected from light. The FITC-conjugated mouse IgG1 antibody clone MOPC-21 was used as an isotype control.

The slides were covered using a DAPI-containing mounting medium (AAT Bioquest, Sunnyvale, CA, USA). Immunofluorescence images were acquired using an Olympus IX71 inverted microscope equipped with an Olympus DP72 digital camera (Olympus Corp., Miami, FL, USA), and image analysis was performed using ImageJ software version 1.50i (NIH, Bethesda, MA, USA).

### 4.8. Ruthenium Red Staining for Evaluation of THP-1 Monocyte Surface Membranes by TEM

Non-infected (Control Mø) and DENV-2-infected THP-1 Mø (DENV-2 Mø) were analyzed by transmission electron microscopy (TEM) using ruthenium red staining to visualize negatively charged surface carbohydrates at the cell membrane, following the protocol described by Martinez-Palomo & Brailovski (1968) [52].

Cell samples were fixed with a solution containing 2.5% glutaraldehyde (EMS, Hatfield, PA, USA) and 50 mg/mL ruthenium red (Sigma-Aldrich) in sodium cacodylate buffer (Sigma-Aldrich) for 1 h at rt, washed three times with sodium cacodylate buffer, and post-fixed with 4% osmium tetroxide (Alfa Aesar, Thermo Fisher Scientific) supplemented with 50 mg/mL ruthenium red for 2 h at rt.

Samples were subsequently dehydrated through a graded ethanol (J.T. Baker) series (50%, 60%, 70%, 80%, 90%, 96%, and 100%) for 5 min each, with the absolute ethanol step repeated three times. Samples were then pre-embedded in a 1:1 mixture of propylene oxide and epoxy resin (Sigma-Aldrich/EMS) for 18 h at rt, embedded in pure epoxy resin, and polymerized at 60 °C for 48 h.

Sections of 40–50 nm thick were mounted onto formvar-coated copper grids (EMS). Sections were contrasted with uranyl acetate (Merck, Darmstadt, Germany) for 30 min and lead citrate (EMS) for 10 min at rt. Observations were performed using a JEOL JEM-1010 transmission electron microscope equipped with a CCD300-RC digital camera (DAGE-MTI; Michigan City, IN, USA). Images were analyzed using ImageJ software.

### 4.9. Isolation of THP-1 Monocyte-Derived MPs by Differential Ultracentrifugation

Culture supernatants from 1.0 × 10^7^ THP-1 monocytes were collected into sterile conical tubes (Corning) and centrifuged at 900× *g* for 10 min at 4 °C to remove intact cells, followed by centrifugation of the recovered supernatants at 2000× *g* for 10 min at 4 °C to eliminate residual cell debris. Clarified supernatants were then transferred to sterile ultracentrifuge tubes (25 × 89 mm; Beckman Coulter, Inc.) and centrifuged at 16,500× *g* for 35 min at 4 °C [53]. Supernatants were discarded, and MP pellets were resuspended in 1.0 mL of PBS at 4 °C. The MPs suspensions were collected into sterile microcentrifuge tubes and used immediately for downstream analyses or stored at −72 °C until use.

### 4.10. Characterization of THP-1 Monocyte-Derived MPs by NTA

The NTA was performed using a NanoSight NS300 system (Malvern Panalytical Products, Mexico City, Mexico) to determine the size distribution and concentration of nanoparticles in THP-1 monocyte-derived EV isolates. Instrument settings were standardized with a camera level of 14, a detection threshold of 2.0, and an operating temperature of 20 °C. MPs Suspensions were diluted 1:50 in PBS, and 1.0 mL of each sample was analyzed. Recordings were acquired for 90 s and measured in triplicate. Polystyrene microspheres of 100 nm (Cat. #NTA4088; Malvern Panalytical Products), diluted 1:500, were analyzed under identical conditions and used as calibration controls.

### 4.11. Morphological Characterization of THP-1 Monocyte-Derived MPs by TEM

THP-1 monocyte-derived MPs preparations were fixed using a solution (1:1) containing 2.5% glutaraldehyde and 4% PFA for 2 h at rt. Fixed samples were subsequently post-fixed with 2% osmium tetroxide for 90 min at rt and washed three times with PBS. Sample dehydration, resin embedding, ultrathin sectioning, contrasting, TEM image acquisition, and image analyses were performed as described in Section 4.8.

### 4.12. Detection of CD14, TF, PS, and Viral E and NS1 Proteins in THP-1 Monocyte-Derived MPs by Flow Cytometry

THP-1 monocyte-derived MPs were fixed with 2% PFA for 10 min at 4 °C, centrifuged at 16,500× *g* for 35 min at 4 °C, and blocked with 2% BSA in PBS for 30 min at rt. MPs samples were subsequently maintained in 0.5% BSA in PBS at 4 °C.

Detection of CD14, TF (CD142), PS, and DENV protein (E and NS1) was performed using the same antibodies, dilutions, incubation conditions, and isotype controls described in Section 4.6. Briefly, CD14 and TF were detected by direct immunostaining using PE-conjugated mouse anti-CD14 and FITC-conjugated mouse anti-CD142 antibodies, respectively. PS exposure was evaluated using FITC-conjugated Annexin V in 1× Annexin V binding buffer, whereas DENV E and NS1 proteins were detected by indirect immunostaining using mouse anti-dengue complex antibody and rabbit anti-DENV NS1 protein, followed by the corresponding fluorophore-conjugated secondary antibodies.

Microbead NIST Traceable Particle Size Standard of 1.00 µm (Cat. #608570; Polysciences, Inc., Warrington, PA, USA) and latex beads of 0.1 µm (Cat. #LB1-1ML; Merck) were used as calibrators and controls. Flow cytometric acquisition was performed using an Attune flow cytometer.

### 4.13. Virion Inactivation in MPs Derived from DENV-Infected THP-1 Monocytes

To reduce residual infectious virions and free viral RNA potentially present in DENV-2-infected THP-1 Mø MPs isolates (DENV-2 Mø MPs), samples were exposed to ultraviolet (UV) radiation at 1200 µJ (×100) for three consecutive cycles using a Stratalinker 1800 system (Stratagene, San Diego, CA, USA). Following UV treatment, MP samples were washed twice with PBS and pelleted by centrifugation at 16,500× *g* for 35 min at 4 °C. UV-treated isolates were used immediately in downstream assays or maintained at 4 °C until use and are hereafter referred to as DENV-2 Mø MPs-UV.

### 4.14. Assessment of Infectious DENV Virions Associated with MPs Isolates by Plaque Assay

To determine whether infectious DENV virions were associated with THP-1 Mø-derived MPs isolates, samples were subjected to UV treatment as described in Section 4.13. Following treatment, MPs were washed twice with PBS, pelleted by centrifugation at 16,500× *g* for 35 min at 4 °C, and resuspended in non-supplemented DMEM. Infectivity was subsequently evaluated by plaque assay as described in Section 4.3. Viral stock and MPs isolated from non-infected monocytes were included as positive and negative controls, respectively.

### 4.15. Thrombin Generation Assay Mediated by THP-1 Monocyte Microparticles

The assay was adapted from the methods described by Nieuwland et al. (2000) and Berckmans et al. (2001) [54,55]. Briefly, thrombin generation was induced by incubating THP-1 monocytes-derived MPs with normal plasma (37 °C) in a buffer A containing 50 mmol/L Tris-HCl (Sigma-Aldrich), 100 mmol/L NaCl (J.T.Baker), and 0.05% BSA (pH 7.35), supplemented with CaCl_2_ (Sigma-Aldrich) to a final concentration of 17 mmol/L. Samples were incubated for 24 h at 37 °C.

Following incubation, reaction aliquots were transferred to buffer B containing 50 mmol/L Tris-HCl, 100 mmol/L NaCl, 29 mmol/L EDTA (J.T.Baker), and 0.05% BSA (pH 7.9) supplemented with 2 mmol/L of the chromogenic substrate S-2238 (Chromogenix S-2238; Cat #S820324; DiaPharma Group Inc., West Chester, OH, USA). The reaction was incubated at 37 °C and stopped after 5 and 20 min using 1 mol/L citric acid (J.T.Baker). Thrombin activity was determined by measuring p-nitroaniline generation at 405 nm (Multiskan Ascent spectrophotometer; Thermo Labsystems, Thermo Fisher Scientific). Data were acquired using Ascent software version 2.6.

### 4.16. Protein Quantification in Isolates of THP-1 Monocyte-Derived MPs by Micro BCA Protein Assay

Protein concentration was determined using the Micro BCA Protein Assay Kit (Thermo Fisher Scientific) according to the manufacturer’s instructions. A calibration curve was generated using 1:2 serial dilutions of a 2 mg/mL BSA standard. Standards and samples were analyzed in duplicate in flat-bottom 96-well microplates (Corning), incubated at 37 °C for 2 h with the working reagent, and absorbance was measured at 562 nm.

### 4.17. Stimulation of Endothelial Vascular Cells (EVC) with THP-1 Monocyte-Derived MPs

Naïve EVC (1.0 × 10^6^ cells per condition) seeded in 25 cm^2^ cell culture flasks (Corning) were stimulated with THP-1 monocyte-derived MPs (Control Mø MPs, DENV-2 Mø MPs, and DENV-2 Mø MPs-UV), normalized to a protein concentration of 1.0 mg/mL. Stimulation was performed in serum-free MCDB-131 medium for 2 h at 37 °C with a 5% CO_2_. Subsequently, culture medium supplemented with 5% EV-depleted FBS was added, and cells were incubated for 72 h under the same conditions. EVC were then harvested using TrypLE Express (Cat. #12604-013; Gibco, Life Technologies Corporation, Thermo Fisher Scientific) and collected by centrifugation at 550× *g* for 10 min at 4 °C for downstream analyses.

### 4.18. Detection of Viral Proteins and Cellular Markers in EVC by Flow Cytometry

THP-1 monocyte-derived MPs-stimulated EVC were fixed with 2% PFA in PBS for 5 min at 4 °C, blocked with 2% BSA in PBS for 30 min at rt, and processed for immunostaining as described in Section 4.6.

For direct immunostaining, FITC-conjugated mouse anti-CD142, FITC-conjugated mouse anti-ICAM-1 (CD54; Cat. #35-0549-T025; Tonbo Biosciences, San Diego, CA, USA), and PE-conjugated mouse anti-VCAM-1 (Cat. #sc-13160; Santa Cruz Biotechnology, Dallas, TX, USA) were used, together with the isotype controls described in Section 4.6.

For indirect immunostaining, mouse anti-protease-activated receptor-1 (PAR-1) (Cat. #SC-13503; Santa Cruz Biotechnology), mouse anti-dengue complex antibody, and rabbit anti-DENV NS1 protein were used as primary antibodies. Immunostaining was completed using the secondary antibodies and isotype controls described in Section 4.6. Flow cytometric analysis was performed using an Attune flow cytometer.

### 4.19. Assessment of EVC Permeability by Transwell Assay

Confluent EVC monolayers (3.5 × 10^5^ cells per condition) were established in sterile polycarbonate transwell inserts (Cat. #3401, Corning) and stimulated with THP-1 monocyte-derived MPs normalized to 1 mg/mL total protein as described in Section 4.17. After 48 h of incubation, endothelial permeability was evaluated by adding FITC–labeled dextran (40 kDa; Cat. #60842-46-8, Sigma-Aldrich), diluted 1:60 in MCDB-131 medium supplemented with 5% EVs-depleted FBS, to the upper chamber and incubating for 1 h at 37 °C.

Media collected from the lower chambers were diluted 1:50 in PBS and transferred to black 96-well plates (Corning). Inserts without cells containing FITC–dextran were included as no-cell controls (NCC) and defined as the 100% permeability reference. Fluorescence intensity was measured at excitation/emission wavelengths of 492/520 nm (Synergy H4 Hybrid Multi-Mode Microplate Reader; BioTek Instruments, Agilent Technologies, Winooski, VT, USA). Data were acquired using Gen5 software version 2.09. Endothelial permeability was expressed as a percentage and calculated as follows: p% = (Fluorescence sample/Fluorescence NCC) × 100.

### 4.20. Statistical Analysis

All experiments were performed independently three to five times. Flow cytometry data were analyzed using FlowJo software version 10 (BD Biosciences). Quantitative results were expressed as mean ± standard deviation, and statistical analyses were performed using GraphPad Prism software version 10.6.1 (GraphPad Software Inc., San Diego, CA, USA).

For comparisons between two independent groups, an unpaired Student’s *t*-test with Welch’s correction was applied. Multiple comparisons among experimental conditions and time points in thrombin generation assays were analyzed using two-way analysis of variance (ANOVA), followed by Tukey’s *post hoc* test with Holm–Šidák correction for multiple testing. For THP-1 monocyte-derived MPs stimulation assays, including permeability assays, differences among groups were evaluated using one-way ANOVA followed by Dunnett’s T3 *post hoc* test. Statistical significance was defined as * *p* < 0.05, ** *p* < 0.01, and *** *p* < 0.0001.

## Data Availability

The original contributions presented in this study are included in the article; further inquiries can be directed to the corresponding author.

## Supplementary Materials

The following supporting information can be downloaded at: https://www.mdpi.com/article/10.3390/ijms27125367/s1.

## Abbreviations

The following abbreviations are used in this manuscript:

CD	Cluster of differentiation
DENV	Dengue virus
DENV E	Dengue virus envelope protein
DENV NS1	Dengue virus nonstructural protein 1
DENV-2 Mø MPs	Microparticles derived from dengue virus-infected monocytes
EVC	Endothelial vascular cells
EVs	Extracellular vesicles
ICAM-1	Intercellular adhesion molecule 1
IL	Interleukin
LPS	Lipopolysaccharide
MPs	Microparticles
Mø	Monocytes
MOI	Multiplicity of infection
mRNA	Messenger ribonucleic acid
NTA	Nanoparticle tracking analysis
TEM	Transmission electron microscopy
TF	Tissue Factor
TNF-α	Tumor necrosis factor alpha
SD	Severe dengue
PAR-1	Protease-activated receptor 1
PS	Phosphatidylserine
VCAM-1	Vascular cell adhesion molecule 1
ZIKV	Zika virus
