# Supplementary material for: Microparticles Released by Dengue Virus-Infected Monocytes Mediate Endothelial Activation and Vasculopathy
Source: Int J Mol Sci. 2026 Jun 14;27(12):5367. doi: 10.3390/ijms27125367 (PMC13299681; doi:10.3390/ijms27125367)
Supplement: Supplementary file 1 [file ijms-27-05367-s001.zip › ijms-4316324-supplementary.pdf]

Supplementary material

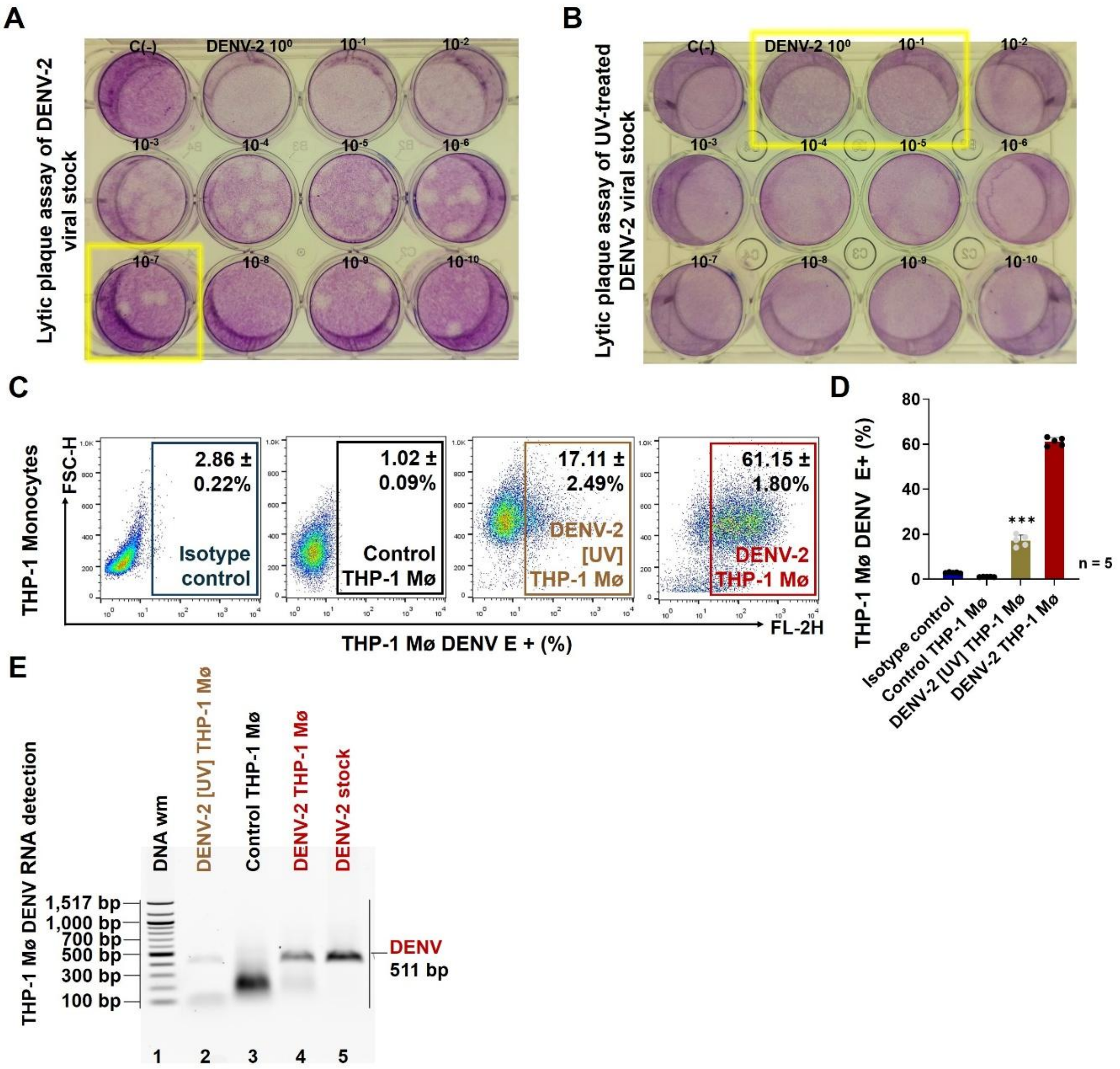

Figure S1. Reduction of viral infectivity through DENV-2 inactivation by UV treatment. (**A**) Lytic plaque assay of DENV-2 viral stock. (**B**) Lytic plaque assay of UV-inactivated DENV-2 viral stock. (**C**) Detection of DENV E protein at 72 h p.i. (representative dot plots by FACS). (**D**) Percentages of E-positive THP-1 Mø. The percentage of THP-1 Mø E+ from DENV-2 [UV] THP-1 Mø was compared with DENV-2 THP-1 Mø value using an unpaired Student's *t*-test. Statistical significance is denoted as *** when $p < 0.0001$. Isotype control (blue), Control THP-1 Mø (black), DENV-2 [UV] THP-1 Mø (brown), and DENV-2 THP-1 Mø / DENV-2 stock (red). n = 5 independent experiments. (**E**) Detection of DENV RNA by RT-PCR amplification. Amplicons were visualized on 1.2% agarose gels stained with 2% ethidium bromide.
